# Endovascular Thrombectomy Versus Best Medical Therapy for Late Presentation Acute Ischemic Stroke With Proximal Large‐Vessel Occlusion Selected on the Basis of Noncontrast Computed Tomography: A Retrospective Analysis of 2 Prospectively Defined Cohorts

**DOI:** 10.1161/SVIN.122.000686

**Published:** 2022-11-18

**Authors:** Permesh Singh Dhillon, Waleed Butt, Tudor G. Jovin, Anna Podlasek, Norman McConachie, Robert Lenthall, Sujit Nair, Luqman Malik, Kailash Krishnan, Iacopo Chiavacci, Farhan Mehedi, Timothy Hong, Harriwin Selva, Robert A. Dineen, Timothy J. England

**Affiliations:** ^1^ Interventional Neuroradiology, Queens Medical Centre Nottingham University Hospitals NHS Trust Nottingham UK; ^2^ NIHR Nottingham Biomedical Research Centre University of Nottingham Nottingham UK; ^3^ Radiological Sciences , Mental Health & Clinical Neuroscience University of Nottingham Nottingham UK; ^4^ Interventional Neuroradiology Queen Elizabeth Hospital University Hospitals Birmingham NHS Trust Birmingham UK; ^5^ Neurology Cooper University Hospital Camden NJ; ^6^ Tayside Innovation Medtech Ecosystem (TIME) University of Dundee UK; ^7^ Stroke Medicine, Queens Medical Centre Nottingham University Hospitals NHS Trust Nottingham UK; ^8^ Stroke, Mental Health and Clinical Neuroscience, School of Medicine University of Nottingham Derby UK; ^9^ Stroke University Hospitals of Derby and Burton NHS Foundation Trust Derby UK

## Abstract

**Background:**

The efficacy and safety of endovascular thrombectomy (EVT) >6 hours from acute ischemic stroke (AIS) onset for patients selected without computed tomography (CT) perfusion or magnetic resonance imaging is undetermined in routine clinical practice.

**Methods:**

In this single‐center study, we identified consecutive late‐presenting patients with AIS who were eligible for EVT on the basis of noncontrast CT/CT angiography (without CT perfusion or magnetic resonance imaging) using an Alberta Stroke Program Early CT Score of ≥6, >6 hours from stroke onset, between January 2018 and March 2022. During the study period, EVT capacity limitations meant EVT‐eligible patients presenting out of regular working hours, consistently received best medical management (BMM). Functional outcomes (modified Rankin Scale at 90 days), symptomatic intracranial hemorrhage, and mortality at 90 days were compared between patients receiving EVT or BMM following multivariable adjustment for age, sex, baseline stroke severity, Alberta Stroke Program Early CT Score, onset‐to‐neuroimaging time, intravenous thrombolysis, and clot location.

**Results:**

Among 4802 patients with AIS, 150 patients (3.1%) presenting beyond 6 hours of onset were eligible for EVT: 74 (49%) treated with EVT and 76 (51%) with BMM. Compared with the BMM group, patients treated with EVT had significantly improved functional outcome (modified Rankin Scale) (adjusted common odds ratio, 2.23 [95% CI, 1.18–4.22]; *P*=0.013), and higher rates of functional independence (modified Rankin Scale ≤2; 39.2.% versus 9.2%; adjusted odds ratio, =4.73 [95% CI, 1.64–13.63]; *P*=0.004). No significant difference was observed between the EVT and BMM groups in the symptomatic intracranial hemorrhage (5.4% versus 2.6%; *P*=0.94) or mortality (20.2% versus 47.3%; *P*=0.16) rates, respectively.

**Conclusion:**

In routine clinical practice, of the 3.1% of patients in our AIS population presenting after 6 hours from stroke onset who were deemed eligible for EVT by noncontrast CT/CT angiography alone, those treated with EVT achieved significantly improved functional outcome, compared with patients treated with BMM only. No significant differences were noted between the 2 groups with respect to symptomatic intracranial hemorrhage and mortality. While confirmatory randomized trials are awaited, these findings suggest that EVT is effective and safe when performed in patients with AIS selected without CT perfusion or magnetic resonance imaging >6 hours from stroke onset.

## Graphical abstract

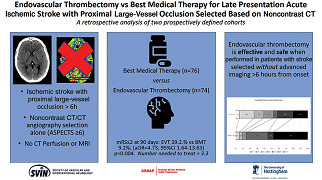

**Nonstandard Abbreviations and Acronyms**
AIS: acute ischemic stroke
ASPECTS: Alberta Stroke Program Early CT Score
BMM: best medical management
CTA: computed tomography angiography
CTP: computed tomography perfusion
DAWN: Diffusion‐Weighted Imaging or Computerized Tomography Perfusion Assessment With Clinical Mimatch in the Triage of Wake‐Up and Late Presenting Strokes Undergoing Neurointervention With Trevo
DEFUSE‐3: Endovascular Therapy Following Imaging Evaluation for Ischemic Stroke
EVT: endovascular thrombectomy
LVO: large‐vessel occlusion
mRS: modified Rankin Scale
NCCT: noncontrast computed tomography
NIHSS: National Institutes of Health Stroke Scale
sICH: symptomatic intracranial hemorrhage

**Clinical Perspective**

**What Is New?**

- We evaluated the functional and safety outcomes of patients with acute ischemic stroke caused by proximal large‐vessel occlusion, presenting >6 hours from stroke onset who were eligible for endovascular thrombectomy (EVT) selected with noncontrast computed tomography/computed tomography angiography alone (without computed tomography perfusion or magnetic resonance imaging), by comparing EVT‐eligible patients treated with EVT (presenting during regular working hours) and a comparable group treated with best medical management only (presenting outside regular working hours).
- Patients treated with EVT achieved significantly improved functional independence, without any significant differences with respect to symptomatic intracranial hemorrhage and mortality, compared with patients treated with best medical management only.
- Our findings suggest that EVT is effective and safe when performed in patients with acute ischemic stroke with proximal large‐vessel occlusion selected without computed tomography perfusion or magnetic resonance imaging >6 hours from stroke onset.

The DAWN (Diffusion‐Weighted Imaging or Computerized Tomography Perfusion Assessment With Clinical Mimatch in the Triage of Wake‐Up and Late Presenting Strokes Undergoing Neurointervention With Trevo) and DEFUSE‐3 (Endovascular Therapy Following Imaging Evaluation for Ischemic Stroke) randomized controlled trials (RCTs) demonstrated benefit of performing endovascular thrombectomy (EVT) for large‐vessel occlusion (LVO) in acute ischemic stroke (AIS) solely for patients selected using advanced neuroimaging (computed tomography perfusion [CTP] or magnetic resonance imaging [MRI]) with a suitable mismatch presenting between 6 and 16 or 24 hours from the onset of stroke or last known well.^1^, ^2^ However, only 1.7%–2.7% of patients presenting with AIS were eligible for EVT on the basis of strict DAWN or DEFUSE‐3 criteria, thereby limiting generalizability of the trials’ favorable findings.^3^ Many institutions have limited access to urgent CTP or MRI and instead, select patients for EVT on the basis of noncontrast CT (NCCT) and CT angiography (CTA). This practice may result in potentially broader and more heterogeneous penumbra–core tissue characteristics compared with trial cohorts.

Given the large positive treatment effect sizes of the late window trials, it is plausible that patients with less favorable imaging profiles may still benefit from EVT.^4^ Although analysis of pooled data from randomized studies of thrombectomy more than 6 hours after last known well (AURORA), a patient level meta‐analysis of all patients randomized between 6 and 24 hours from stroke onset, included patients selected on the basis of NCCT/CTA, definitive proof of EVT treatment could not be demonstrated between 6 and 12 hours from stroke onset as the analysis was underpowered.^5^ Ongoing RCTs are assessing whether treatment benefit with EVT is maintained in patients presenting >6 hours of stroke onset (late window) when less restrictive clinical and imaging selection criteria are used.^6^, ^7^ In the interim, various nonrandomized studies have attempted to assess the functional and safety outcome data following EVT in the absence of CTP or MRI in the late window.^8^, ^9^, ^10^, ^11^, ^12^, ^13^ However, the assessment of eligibility for EVT and absolute treatment efficacy or benefit in this window has been limited because of the lack of comparison to a control group of patients who did not undergo EVT.

Hence, we sought to evaluate the incidence of patients with AIS presenting >6 hours from stroke onset who were eligible for EVT selected with NCCT/CTA alone (without CTP or MRI) and their functional and safety outcomes by comparing EVT‐eligible patients treated with EVT and a comparable group with respect to baseline characteristics treated with best medical management (BMM) only. Limitations in EVT capacity at our institution during the study period meant that by default (and with no exceptions) patients presenting on weekdays between 6 pm and 8 am (out of regular work hours) or on weekends were unable to receive EVT and were treated with BMM. This systemic unavailability of EVT allows a comparison of EVT and BMM in patients presenting >6 hours from onset who meet the same inclusion criteria, in which selection based on physician‐related bias is significantly reduced.

## Methods

### Ethics

This study was registered with and approved by the local institutional board review (Ref ID: 22‐158C). Retrospective patient consent was not required for this study, which was conducted in a deidentified manner. Data that support the findings of this study are available upon reasonable request.

### Data Source and Study Design

We performed a retrospective analysis of 2 prospectively defined cohorts according to the Strengthening the Reporting of Observational Studies in Epidemiology guidelines, on prospectively collected Sentinel Stroke National Audit Programme registry data^11^ for all adult (aged ≥18 years) consecutive admissions with an AIS who presented directly to a single tertiary EVT‐capable neuroscience center in the United Kingdom, between January 1, 2018, and March 31, 2022. The selection of EVT‐eligible patients in clinical practice was based on our institution's protocol of the initial imaging performed (NCCT and/or dual‐phase CTA) regardless of the time window from stroke onset. The inclusion criteria for this study included (1) occlusion of the intracranial internal carotid artery or M1 segment of the middle cerebral artery, (2) premorbid disability using the modified Rankin Scale (mRS) of 0 to 2, (3) baseline stroke severity National Institutes of Health Stroke Scale (NIHSS) of ≥6, (4) baseline Alberta Stroke Program Early CT Score (ASPECTS) of ≥6, and (5) presentation from stroke onset or last known well to neuroimaging time of >6 hours. Patients with a stroke onset to neuroimaging time of <6 hours, those with an ASPECTS score of 0 to 5, posterior circulation stroke, or medium/distal vessel occlusions were excluded. The mRS data at discharge were extrapolated (assuming no further improvement or worsening) for those with missing 90‐day mRS scores (n=4).

Patients were divided into 2 groups according to the treatment received: (1) EVT and (2) BMM (with or without intravenous thrombolysis). At our institution over the study period, EVT treatment was offered to eligible patients only between 8 am and 6 pm Monday through Friday because of limited capacity. Patients who presented out of hours or on weekends, including those eligible for EVT treatment, were treated with BMM only. Because of the regional service limitations during the study period, patients could not be transferred to another center offering EVT out of regular hours. A small cohort of patients who presented out of hours but remained eligible the next day following repeat neuroimaging were offered EVT treatment. A minority of patients may have also presented <6 hours from stroke onset (out of hours) and were treated with intravenous tissue plasminogen activator, but remained eligible for EVT treatment following repeat neuroimaging in the late window. Patients who received BMM at our institution were admitted to a dedicated hyperacute stroke unit and were treated according the National Institute for Health and Care Excellence guidelines,^14^ which included 300 mg of aspirin on admission if they were ineligible for intravenous tissue plasminogen activator treatment, adequate blood pressure, and blood glucose control.

### Outcome Measures

The main functional outcome was assessed with the mRS score at 90 days, ranging from 0 (no symptoms) to 5 (severe disability/bedridden) and 6 (death). Other functional outcomes were functional independence (mRS ≤2) and excellent (mRS ≤1) functional outcome at 90 days. Safety outcomes were mortality at 90 days and symptomatic intracranial hemorrhage (sICH) defined according to European Collaborative Acute Stroke Study II classification^15^ as any intracranial hemorrhage with an increase of the NIHSS score of ≥4 within 24 hours or death. Procedural outcomes were successful reperfusion modified thrombolysis in cerebral infarction score of 2b–3 (50%–100% vascular territory reperfusion) at the end of EVT.^16^ Workflow time metrics were stroke onset to neuroimaging, stroke onset to arterial puncture, and total procedural time (arterial puncture to final angiographic run). Baseline clinical data were retrieved from the prospective stroke registry and functional outcome measure (mRS) was prospectively assessed by a member of the stroke team/physician at routine clinical follow‐up or by a trained specialist nurse during a follow‐up telephone interview if the patient was unable to attend. ASPECTS and collateral circulation status were retrospectively assessed by 2 trained neuroradiologists, with disagreements resolved by a third neuroradiologist, all blinded to the treatment allocation. Intraclass correlation coefficient was calculated as a measure of interrater reliability between the ASPECTS obtained prospectively for treatment purposes and the ASPECTS following retrospective review.

### Statistical Analysis

Study characteristics were summarized using descriptive statistics for patient demographics, clinical characteristics, comorbidities, and time metrics. Comparisons of baseline variables were made using the chi‐square, or Student's *t*‐test, wherever applicable.

Analyses of the outcome measures used ordinal logistic regression for the full‐scale mRS as primary outcome and binary regression analysis for the remaining dichotomized clinical outcomes. Multivariable regression analysis was conducted, adjusted for variables of clinical relevance: age, sex, baseline stroke severity (NIHSS), baseline ASPECTS, clot location, stroke onset‐to‐neuroimaging time, and prior intravenous tissue plasminogen activator use.

A prespecified subgroup analysis similar to the AURORA analysis^5^ was performed comparing the (1) age (≤70 years versus >70 years), (2) sex, (3) patients treated in the 6‐ to 12‐hour and >12‐hour time windows, (4) clot location (internal carotid artery versus middle cerebral artery), (5) baseline NIHSS (≤17 versus >17), (6) baseline ASPECTS (6–7 and 8–10), and (7) according to the stroke presentation (witnessed versus wake‐up/last known well). The interpretation of any subgroup effect was based on interaction tests. The absolute risk reduction and number needed to treat (NNT) to achieve functional independence at 90 days were evaluated. A 2‐tailed *P* value of <0.05 was considered statistically significant. Analyses were conducted using StataSE 17.1 (StataCorp, College Station, TX) and R software 4.2.1 (R Foundation for Statistical Computing, Vienna, Austria).

## Results

### Characteristics of Study Population

**Figure 1:**
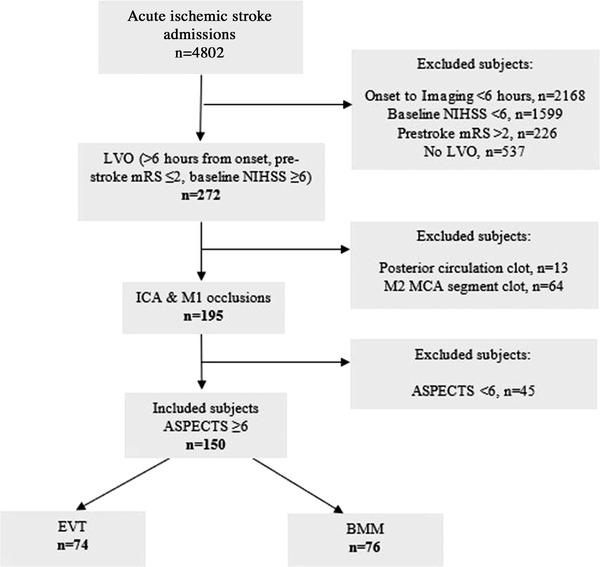
**Flowchart of the patient inclusion, exclusion, and outcome data for patients identified** **as eligible for EVT without CTP or magnetic resonance neuroimaging and treated with EVT or BMM beyond 6 hours from stroke onset or last known well**. ASPECTS indicates Alberta Stroke Program Early Computed Tomography Score; BMM, best medical management; CTP, computed tomography perfusion; EVT, endovascular thrombectomy; ICA, internal carotid artery; LVO, large‐vessel occlusion; MCA, middle cerebral artery; mRS, modified Rankin scale; and NIHSS, National Institutes of Health Stroke Scale.

During the study period, a total of 4802 patients with AIS presented directly to a single EVT‐capable neuroscience center. Of these patients, 4652 who did not meet the inclusion criteria of the study were excluded (Figure 1). We included 150 patients presenting after 6 hours and assessed as being eligible for EVT (3.1% of all AIS admissions), of whom 74 (49%) were treated with EVT, and 76 (51%) were treated with BMM only. Sixty patients (40%) had a witnessed stroke onset, and the remainder were documented as last known well/wake‐up. Compared with the BMM cohort, patients treated with EVT were younger (68.5±14.8 versus 77.1±13.9 years) and had a lower baseline stroke severity (NIHSS) (median 18 [12, 13, 14, 15, 16, 17, 18, 19, 20, 21, 22] versus 19 [16, 17, 18, 19, 20, 21, 22, 23, 24, 25]) (Table 1). No significant differences were observed in the remaining baseline characteristics, including the baseline ASPECTS (median 7 [6, 7, 8] versus 7 [6.75–8]), between the 2 groups (Table 1). The mean time to neuroimaging in the EVT cohort was 866.5±533.0 minutes compared with 964.5±563.6 minutes in the BMM group. No EVT‐eligible patients were treated with EVT outside of the regular work hours during the study period. There was good interrater reliability (intraclass correlation coefficient=0.677 [95% CI, 0.554–0.800]) between the ASPECTS obtained for treatment purposes and following the retrospective review.

**Table 1:** Table of Characteristics Comparing Patients Identified as Eligible for EVT Without CTP or Magnetic Resonance Neuroimaging and Treated With EVT or BMM >6 Hours from Stroke Onset or Last Known Well

Feature	EVT, n (%) median (IQR) or mean±SD	BMM, n (%) median (IQR) or mean±SD	*P* value
Sociodemographics			
Sample size	74	76	–
Sex, male	41 (55.4)	34 (44.7)	0.19
Age, y	68.5±14.8	77.1±13.9	**0.004**

Baseline characteristics			
NIHSS on admission	18 (12, 13, 14, 15, 16, 17, 18, 19, 20, 21, 22)	19 (16, 17, 18, 19, 20, 21, 22, 23, 24, 25)	**0.006**
Prestroke disability (mRS)	0 (0–0)	0 (0–0)	0.10
ASPECTS	7 (6, 7, 8)	7 (6.75–8)	0.83
ICA clot	20 (27.0)	20 (26.3)	0.92
M1 clot	54 (73.0)	56 (73.6)	0.92
Moderate‐good collaterals (Tan 2–3)*	59 (79.7)	26/33 (78.7)	0.50
IV thrombolysis	6 (8.1)	12 (15.7)	0.15
Witnessed stroke onset	28 (37.8)	32 (42.1)	0.59

Comorbidities			
Hypertension	35 (47.3)	42 (55.3)	0.33
Diabetes	18 (24.3)	11 (14.5)	0.13
Atrial fibrillation	14 (18.9)	19 (25.0)	0.37
Prior stroke/TIA	9 (12.1)	13 (17.1)	0.39

Time metrics (min)			
Onset to neuroimaging	866.5±533.0	964.5±563.6	0.27
Onset to arterial puncture	966.4±523.4	–	–
Arterial puncture to end of procedure	61.8±32.9	–	–

ASPECTS indicates Alberta Stroke Program Early Computed Tomography Score; BMM, best medical management; EVT, endovascular thrombectomy; ICA, internal carotid artery; IQR, interquartile range; IV, intravenous; M1, first segment of the middle cerebral artery; mRS, modified Rankin scale; and NIHSS, National Institutes of Health Stroke Scale; and TIA, transient ischemic attack.

* Available data: N=33 BMM group.

### Outcomes

**Figure 2:**
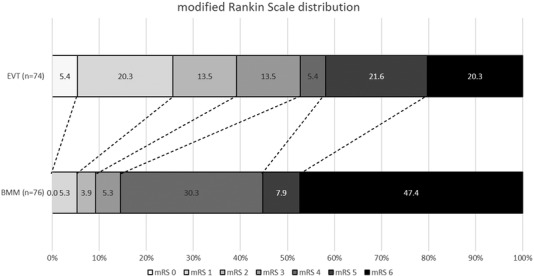
**Distribution of the modified Rankin scale (0 – no disability to 5 – severe disability and 6 – death)** **at 90 days comparing patients with large‐vessel occlusion selected without CTP or magnetic resonance neuroimaging and treated with EVT or BMM beyond 6 hours from stroke onset or last known well**. BMM indicates best medical management; CTP, computed tomography perfusion; and EVT, endovascular thrombectomy.

**Figure 3:**
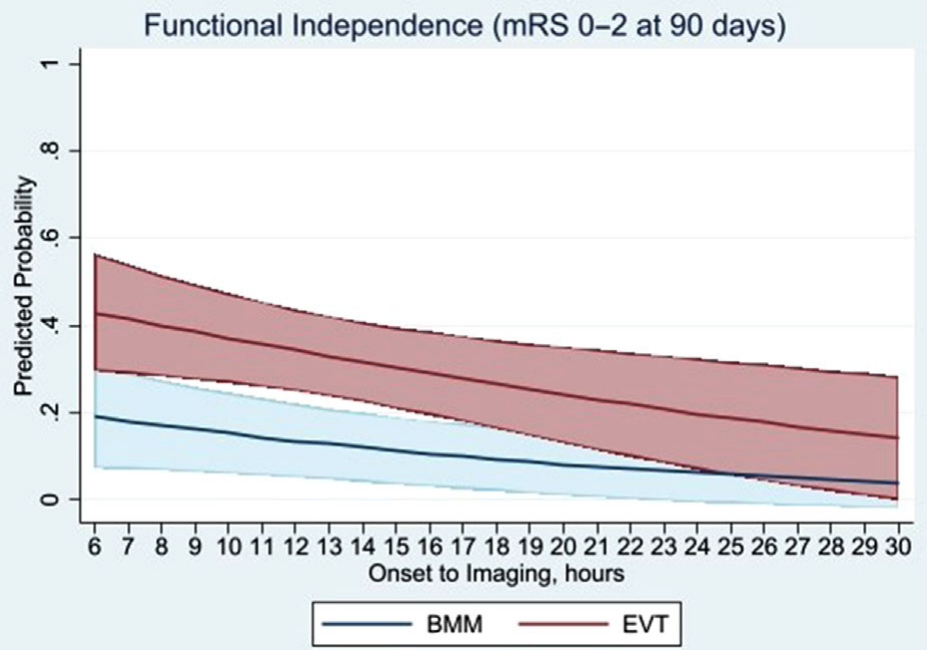
**Association between stroke onset‐to‐neuroimaging time >6 hours among** **EVT and BMM patients with large vessel occlusion in acute ischemic stroke and the functional independence (mRS ≤2 at 90 days)**. Analyses used time as a continuous variable in minutes and were adjusted for age, sex, baseline NIHSS, ASPECTS, clot location, and use of intravenous thrombolysis. The central line indicates the predicted outcomes for a hypothetical patient with mean values for the adjusted baseline characteristics and the shaded area represents the 95% CIs. ASPECTS indicates Alberta Stroke Program Early Computed Tomography Score; BMM, best medical management; EVT, endovascular thrombectomy; mRS, modified Rankin scale; and NIHSS, National Institutes of Health Stroke Scale.

Compared with the BMM group, patients selected with NCCT/CTA alone and treated with EVT >6 hours from stroke onset had significantly greater odds of improving the mRS score by 1 point at 90 days (adjusted common odds ratio=2.23 [95% CI, 1.18–4.22]; *P*=0.013), and achieved significantly higher rates of functional independence (mRS ≤2 at 90 days; 39.2.% versus 9.2%; adjusted odds ratio=4.73 [95% CI, 1.64–13.63]; *P*=0.004), and excellent functional outcome (mRS ≤1 at 90 days; adjusted odds ratio=5.32 [95% CI, 1.42–19.82]; *P*=0.013) (Table 2, Figures 2 and 3). The absolute risk reduction and NNT for functional independence at 90 days were 30% and 3.3, respectively. However, no statistically significant difference was observed between the EVT and BMM groups in the remaining outcomes measures of sICH (5.4% versus 2.6%; *P*=0.94) or mortality at 90 days (20.2% versus 47.3%; *P*=0.16), respectively (Table 2).

**Table 2:** Table of Outcomes Comparing Patients Identified as Eligible for EVT Without CTP or MR Neuroimaging and Treated With EVT or BMM >6 Hours From Stroke Onset or Last Known Well

			EVT versus BMM			
Outcome measures	EVT (n=74) N (%)	BMM (n=76) N (%)	Unadjusted OR (95% CI)	*P* value	Adjusteda OR (95% CI)^†^	*P* value
mRS at 90 d (ordinal)	3 (1.25–5)	5 (4, 5, 6)	3.57 (1.96–6.50)	0.0001*	2.23 (1.18–4.22)	0.013*
mRS ≤1	19 (25.6)	4 (5.2)	6.21 (2.00–19.32)	0.002*	5.32 (1.42–19.82)	0.013*
mRS ≤2	29 (39.2)	7 (9.2)	6.35 (2.56–15.73)	0.0001*	4.73 (1.64–13.63)	0.004*
mTICI 2b‐3	71 (95.9)	–	–	–	–	–
sICH	4 (5.4)	2 (2.6)	2.11 (0.37–11.91)	0.39	1.04 (0.15–6.96)	0.96
Mortality (90 d)	6 (20.2)	36 (47.3)	0.28 (0.13–0.58)	0.001*	0.53 (0.22–1.29)	0.16

aOR indicates adjusted odds ratio; ASPECTS, Alberta Stroke Program Early CT Score; BMM, best medical management; CTP, computed tomography perfusion; EVT, endovascular thrombectomy; mRS, modified Rankin scale; mTICI, modified thrombolysis in cerebral infarction; NIHSS, National Institutes of Health Stroke Scale; OR, odds ratio; and sICH, symptomatic intracranial hemorrhage. ^*^Statistically significant.

^†^ Adjusted multivariate analysis for age, sex, baseline NIHSS, ASPECTS, onset‐to‐imaging time, use of intravenous thrombolysis, and clot location.

**Figure 4:**
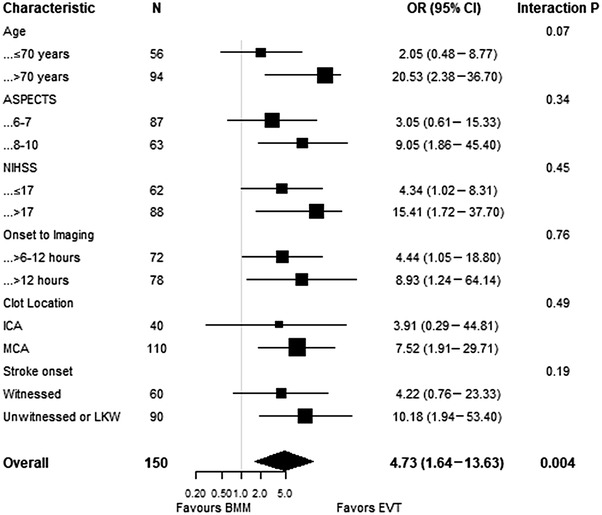
**Forest plot of prespecified subgroups (based on the modified Rankin Scale ≤2 at 90 days)** **of patients with large vessel occlusion selected without CTP or magnetic resonance neuroimaging beyond 6 hours from stroke onset or last known well and treated with EVT or BMM**. ASPECTS indicates Alberta Stroke Program Early CT Score; BMM, best medical management; CTP, computed tomography perfusion; EVT, endovascular thrombectomy; ICA, internal carotid artery; LKW, last known well; MCA, middle cerebral artery (first segment M1); NIHSS, National Institutes of Health Stroke Scale; and OR, odds ratio.

Subgroup comparisons of both EVT and BMM cohorts within the 6–12 hour and >12 hour windows demonstrated a significant treatment effect in favor of EVT in both time windows, without evidence of treatment interaction (*P*=0.76) (Figure 4). EVT remained effective in most subgroups, except in patients with a younger age (≤70 years), lower ASPECTS of 6 to 7, internal carotid artery occlusion, and witnessed stroke onset (Figure 4). No significant treatment interaction was observed in all subgroups.

## Discussion

This study provides novel real‐world data on the eligibility of patients for EVT and the clinical outcomes, among those who were eligible for EVT and treated with EVT compared with BMM only, following imaging selection with NCCT/CTA only (without CTP or MRI) >6 hours from stroke onset. Compared with patients treated with BMM only, significantly improved functional outcome and functional independence (mRS ≤2) at 90 days (39.2% versus 9.2%) were observed in patients treated with EVT. The incidence of sICH and mortality rate at 90 days were numerically higher in the EVT group and BMT group, respectively, than in their respective comparison group, but the difference was not significant following adjustment for confounders.

Previous studies have attempted to indirectly compare advanced imaging (CTP or MRI) and nonadvanced imaging (NCCT/CTA alone) selection modalities >6 hours from onset, results of which have largely demonstrated no difference in functional outcome following EVT when either imaging technique is used in the late window.^13^, ^17^ However, the lack of a control group of patients treated with BMM in the previous studies has restricted the assessment of the absolute treatment efficacy and eligibility for EVT among patients selected with NCCT/CTA alone. Furthermore, the recent AURORA pooled analysis of individual patient data of RCTs failed to demonstrate a statistically significant treatment benefit of EVT in patients selected without an imaging profile determined by CTP or MRI in the late window, possibly because the sample size was modest (n=132).^5^, ^18^ Our findings suggest that the use of NCCT/CTA alone might be a feasible option to select patients for EVT >6 hours from onset, as evidenced by the 30‐percentage‐point difference in the functional independence rate with net treatment benefit and comparable safety outcomes between the EVT and BMM groups in the late EVT window, a treatment effect size that is comparable to the one noted in DAWN and DEFUSE 3. Furthermore, the NNT to achieve functional independence at 90 days in our study was 3.3, which was in line with that reported in the DAWN (NNT=2.8) and DEFUSE‐3 (NNT=3.6) trials.^1^, ^2^ Although some of the subgroup analyses in our study were likely underpowered, a treatment effect in favor of EVT was still present >12 hours from stroke onset. Furthermore, based on the association between time to neuroimaging and the functional independence analysis, patients treated with EVT had a significantly improved outcome compared with the BMM group from 6 up to at least 17 hours from stroke onset, beyond which overlap in the wide CIs may indicate the sample size was inadequate to detect further significant associations (Figure 3). Our results are consistent with the ones noted in AURORA, where the magnitude of treatment effect was greater in the 12‐ to 24‐hour group than in the 6‐ to 12‐hour group. Overall, considering the large positive treatment effect sizes observed in the late‐window RCTs, it is conceivable that patients with a less favorable imaging profile would still benefit from EVT.^4^ Hence, the results of the ongoing RCTs selecting patients within the 6‐ to 24‐hour window without CTP or MRI are eagerly awaited.^6^, ^7^

The rate of functional independence in the EVT arm of our study (39.2%) was comparable to that reported in the recent AURORA pooled analysis of individual patient data of RCTs, which included the DAWN and DEFUSE‐3 late‐window trials (45.9%). The incidence of sICH and mortality in our EVT arm were similar to the DAWN and DEFUSE‐3 trials. Previous observational studies have also reported a range of functional independence rates, ranging from 20% to 64% using various prespecified clinical and imaging patient selection criteria in the late EVT window.^8^, ^9^, ^10^, ^11^, ^12^, ^13^, ^19^, ^20^, ^21^, ^22^, ^23^, ^24^ Some of these investigations incorporated perfusion‐based imaging with varying adherence to the DAWN and DEFUSE‐3 eligibility criteria,^19^, ^20^, ^21^, ^22^, ^23^ while others used solely NCCT and CTA but also varied in their selection criteria.^8^, ^9^, ^10^, ^12^, ^13^ Although advanced imaging (CTP or MRI) may reliably select “slow progressors” who have a higher chance of achieving functional independence following EVT compared with “fast progressors” in the late window,^25^, ^26^ our findings suggest that an equally likely high proportion of “slow progressors” with a limited infarct core and good collateral supply may also be feasibly selected with NCCT/CTA alone throughout the late window. This observation is supported by previous reports of increased sensitivity of NCCT compared with CTP in detecting early ischemic change in the late time window.^27^ Furthermore, a previous study also reported that 79% of patients with anterior circulation LVO with an ASPECTS ≥6 and arriving >6 hours from stroke onset met the DAWN‐defined clinical‐imaging mismatch threshold for EVT eligibility.^28^

The American Heart Association/American Stroke Association and the European Society of Minimally Invasive Neurological Therapy guidelines recommend the use of strict CTP‐ or MRI‐based imaging criteria used in the DAWN and DEFUSE‐3 trials for patient selection for EVT in the late time window.^29^, ^30^ However, because of limited access to urgent CTP or MRI, many institutions in the United Kingdom and various parts of the world use more widely available NCCT/CTA imaging only in routine clinical practice to estimate the infarct size (ASPECTS) and collateral supply irrespective of the time window, thereby impeding the adherence to such guidelines. In addition, the time penalty associated with advanced imaging acquisition and increased radiation exposure warrant simplification of the patient selection process for EVT. Furthermore, the recent contrast shortage in the United States highlights the need for alternative simplified selection paradigms even in health care systems equipped with advanced imaging capabilities.^31^

Direct comparisons regarding superiority of imaging modality selection are difficult and subject to a denominator bias given the varying clinical inclusion criteria across studies^32^ and that we included only patients without advanced (CTP or MRI) imaging. In our study, 3.1% (150/4802) of all consecutive AIS admissions who presented directly to a comprehensive stroke center were eligible for EVT treatment using NCCT/CTA imaging selection alone (Figure 1), which was higher than the reported estimate (1.7%–2.7%) in a single comprehensive stroke center that employed the DAWN or DEFUSE‐3 trial criteria for patient selection.^3^ A total of 57.9% (150/259) of all late‐presenting patients with anterior circulation LVO were eligible for EVT in our study population (Figure 1), which was also higher than that observed (29.0%) in the previous study.^3^ It is noteworthy that, although more stringent imaging trial criteria using CTP or MRI may lead to a marginally higher likelihood of an individual patient achieving functional independence, the resulting smaller proportion of patients eligible for EVT limits the potential treatment impact on the population as a whole.

The main strength of this study is that it allowed a comparison of 2 treatment approaches between equivalent study populations without physician influence with regard to treatment allocation. This contention is supported by the fact that the policy of no treatment during the established hours was pursued without exception and minimizes selection bias, a major confounder in real‐world retrospective analyses comparing EVT with BMM for LVO stroke. There are several limitations of this study. First, because of its observational design, confounding by indication and selection bias may have influenced the results. It is possible that there are inherent differences in the patient populations with LVO stroke presenting during regular hours versus outside of regular hours, although we are not aware of studies demonstrating such differences. Second, there were some differences in between‐group baseline characteristics likely attributable to the small number of patients in both groups and the inclusion of a small cohort of patients (n=16) in the EVT group who presented out of hours but remained eligible and were offered EVT treatment the next day following repeat neuroimaging. To mitigate confounding, these baseline variables were adjusted for in multivariable analyses. Third, there were some missing data for the collateral circulation assessment in the BMM cohort, as a small group (28%) of patients did not undergo CTA imaging on arrival. Fourth, the wide CIs in the late EVT window may indicate that the modest sample size was inadequate to detect significant associations particularly >17 hours from stroke onset or last known well, and that the subgroup analyses might be underpowered. Fifth, our study included only patients selected with NCCT/CTA alone, and hence, comparisons with CTP or MRI and estimation of infarct volumes could not be assessed. Finally, the outcome measures, although collated prospectively, were not independently evaluated by a core laboratory.

## Conclusions

In this study, patients selected with NCCT/CTA alone and treated with EVT >6 hours from stroke onset achieved significantly higher rates of functional independence at 90 days, with comparable rates of sICH and mortality, compared with patients eligible for EVT but treated with BMM only. A total of 3.1% of patients with AIS were eligible for EVT using more liberal clinical and imaging selection criteria on the basis of NCCT/CTA alone in the late window. While confirmatory randomized trials are awaited, these findings suggest that EVT is effective and safe when performed in patients with AIS selected without CTP or MRI >6 hours from stroke onset or last known well in routine clinical practice.

## Author Contributions

Conception and design and Acquisition of the data: Permesh Singh Dhillon. Analysis and interpretation of the data: Permesh Singh Dhillon, Waleed Butt, Iacopo Chiavacci, Farhan Mehedi, Timothy Hong, and Harriwin Selva. Critical revision of the manuscript: Permesh Singh Dhillon, Waleed Butt, Tudor G. Jovin, Anna Podlasek, Norman McConachie, Robert Lenthall, Sujit Nair, Luqman Malik, Kailash Krishnan, Robert A. Dineen, and Timothy J. England. Study supervision: Robert A. Dineen and Timothy J. England. All authors approved the final version of the manuscript.

## Sources of Funding

No specific funding was sought for this study.

## Disclosures

Tudor G. Jovin is advisor and investor for Anaconda, Route92, Viz.AI, FreeOx, Blockade Medical, and Methinks. He received personal fees in his role on the Data Safety Monitoring Board and steering committee from Cerenovus and on the screening committee for Contego Medical. He received stock as an advisory board member for Corindus. He received grant support from Medtronic and from Stryker Neurovascular in his capacity as principal investigator for DAWN and AURORA. The remaining authors declare no other disclosures or competing interests.
